# Supplementary material for: Phytochemical Composition and Biological Properties of Macleania rupestris Fruit Extract: Insights into Its Antimicrobial and Antioxidant Activity
Source: Antioxidants (Basel). 2025 Mar 27;14(4):394. doi: 10.3390/antiox14040394 (PMC12024342; doi:10.3390/antiox14040394)
Supplement: Supplementary file 1 [file antioxidants-14-00394-s001.zip › antioxidants-3526959-supplementary.pdf]

Supplementary material

Phytochemical Composition and Biological Properties of *Macleania rupestris* Fruit Extract: Insights into Its Antimicrobial and Antioxidant Activity

Arianna Mayorga-Ramos [1], Johana Zúñiga-Miranda [1], Elena Coyago-Cruz [2], Jorge Heredia-Moya [1], Jéssica Guamán-Bautista [3] and Linda P. Guamán [1,*]

[1] Centro de Investigación Biomédica, Facultad de Ciencias de la Salud Eugenio Espejo, Universidad UTE, Quito 170129, Ecuador; arianna.mayorga@ute.edu.ec (A.M.-R.); johana.zuniga@ute.edu.ec (J.Z.-M.); jorgeh.heredia@ute.edu.ec (J.H.-M.)

[2] Carrera de Ingeniería en Biotecnología de los Recursos Naturales, Universidad Politécnica Salesiana, Sede Quito, Campus El Girón, Av. 12 de Octubre N2422 y Wilson, Quito 170143, Ecuador; ecoyagoc@ups.edu.ec

[3] Facultad de Ciencias de la Hospitalidad, Carrera de Gastronomía, Universidad de Cuenca, Cuenca 010201, Ecuador; jessica.guaman@ucuenca.edu.ec

* Correspondence: linda.guaman@ute.edu.ec

Figure S1. *Macleania rupestris* (Kunth) A.C.Sm.

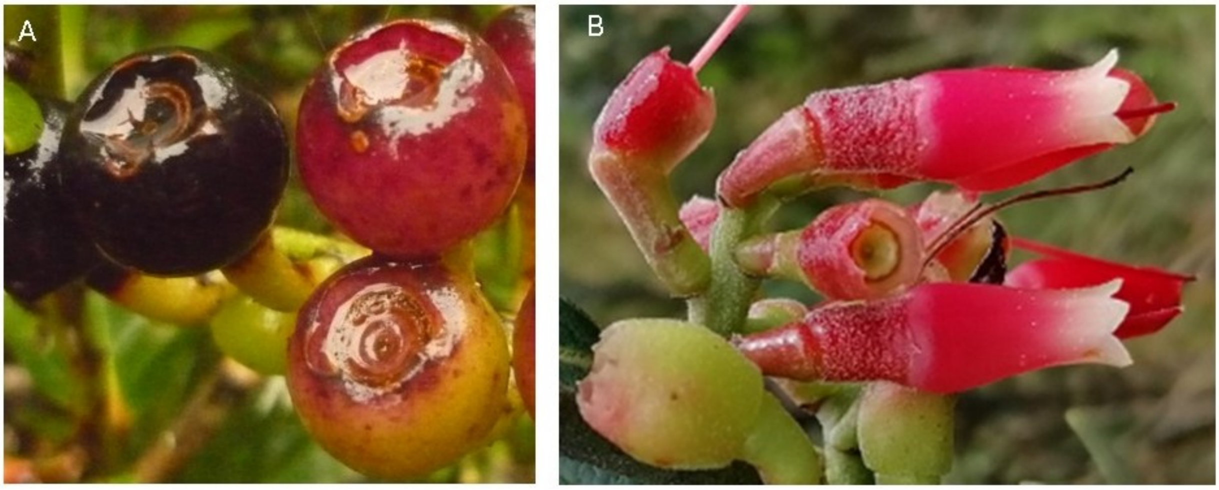

Figure S2. Botanical identification of *Macleania rupestris*

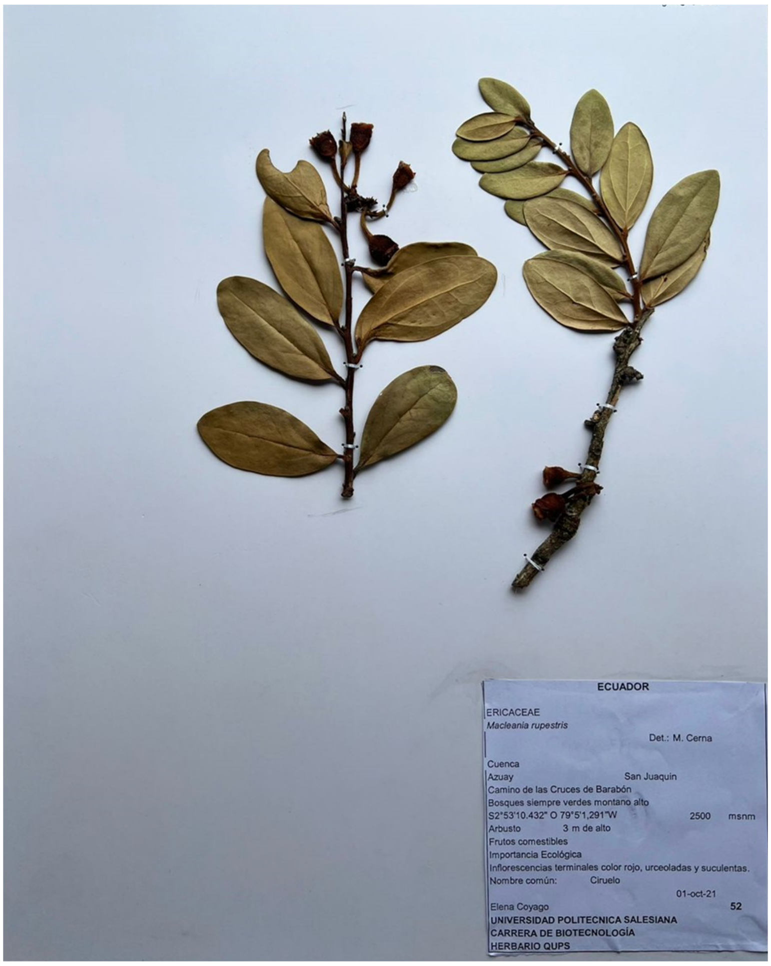

Table S1. Resistance profile of the bacterial strains used during the antibacterial activity evaluation

**Bacterial strain**	**Resistance**
Klebsiella pneumoniae	Serine carbapenemase, Colistin resistance
Escherichia coli	Serine carbapenemase, (Metallo β-lactamase)
Enterococcus faecalis	Vancomycin resistance -VAN
Staphylococcus epidermidis	Vancomycin resistance -VAN
Enterococcus faecium	Vancomycin resistance -VAN
Salmonella enterica serovariedad Kentucky	Metallo β-lactamase resistance to azithromycin
Pseudomona aeruginosa	BLEE+ Impermeability

Table S2. Antifungal activity of *M. rupestris* extract (Joyapa) against *Candida* species

Extract concentration (mg/mL)	*C. albicans*	*C. glabrata*	*C. krusei*	*C. tropicalis*
**20**	N-Inh	N-Inh	N-Inh	N-Inh
**10**	N-Inh	N-Inh	N-Inh	N-Inh
**5**	N-Inh	N-Inh	N-Inh	N-Inh
**2.5**	N-Inh	N-Inh	N-Inh	N-Inh
**0.5**	N-Inh	N-Inh	N-Inh	N-Inh
**0**	N-Inh	N-Inh	N-Inh	N-Inh

N-Inh: No inhibition of microorganism growth at the described concentration

Table S3. Percentage of Biofilm Inhibition Activity of *M. rupestris* extract.

Extract concen-tration	*Staphylococcus aureus* ATCC 25923		*Listeria mono-cytogenes* ATCC 13932		*Pseudomonas aeruginosa* ATCC 9027		*Burkholderia ce-pacia* ATCC 25416	
**(ug/mL)**	**Mean**	**SD**	**Mean**	**SD**	**Mean**	**SD**	**Mean**	**SD**
**30**	78%	1.86%	73%	2.46%	71%	2.66%	97%	0.11%
**20**	79%	2.16%	77%	4.54%	75%	2.15%	98%	0.46%
**10**	81%	2.44%	83%	2.59%	76%	2.91%	99%	0.43%
**5**	77%	3.11%	69%	7.64%	N-Inh	-	91%	0.72%
**1**	71%	3.37%	72%	8.63%	N-Inh	-	69%	7.69%
**0.1**	36%	6.75%	N-Inh	-	4%	7.51%	N-Inh	-

N-Inh: No inhibition of microorganism biofilm growth at the described concentration
